# Breaking down barriers in morning glories

**DOI:** 10.1111/mec.15048

**Published:** 2019-04-09

**Authors:** David L. Field, Christelle Fraïsse

**Affiliations:** ^1^ Department of Botany and Biodiversity Research, Faculty of Life Sciences University of Vienna Vienna Austria; ^2^ Institute of Science and Technology Austria Klosterneuburg Austria

**Keywords:** hybridization, molecular evolution, plant mating systems, population genetics – empirical, speciation, transcriptomics

## Abstract

One of the most striking and consistent results in speciation genomics is the heterogeneous divergence observed across the genomes of closely related species. This pattern was initially attributed to different levels of gene exchange—with divergence preserved at loci generating a barrier to gene flow but homogenized at unlinked neutral loci. Although there is evidence to support this model, it is now recognized that interpreting patterns of divergence across genomes is not so straightforward. One problem is that heterogenous divergence between populations can also be generated by other processes (e.g. recurrent selective sweeps or background selection) without any involvement of differential gene flow. Thus, integrated studies that identify which loci are likely subject to divergent selection are required to shed light on the interplay between selection and gene flow during the early phases of speciation. In this issue of *Molecular Ecology*, Rifkin et al. (2019) confront this challenge using a pair of sister morning glory species. They wisely design their sampling to take the geographic context of individuals into account, including geographically isolated (allopatric) and co‐occurring (sympatric) populations. This enabled them to show that individuals are phenotypically less differentiated in sympatry. They also found that the loci that resist introgression are enriched for those most differentiated in allopatry and loci that exhibit signals of divergent selection. One great strength of the study is the combination of methods from population genetics and molecular evolution, including the development of a model to simultaneously infer admixture proportions and selfing rates.

In general, the separation of species requires the evolution of genetic barriers to gene exchange that are strong enough to overpower the homogenizing effects of gene flow. Theory predicts that genomic heterogeneity in divergence can be produced by interspecific barriers that act locally in the genome to restrict gene flow (Barton & Bengtsson, 1986). Given the relative ease in which genome‐wide data can now be obtained, genome scans are routinely used between phenotypically divergent populations to identify regions with strong allele frequency differences or excess relative divergence (*F*_ST_). Although the limitations of these methods for signalling differential gene flow has been recognized for some time (Charlesworth, 1998), the complications with genome scans were not widely appreciated until the number of data sets began to accumulate (Cruickshank & Hahn, 2014).

A major pitfall of these genomic studies is that chromosomal features (recombination rates) and population processes (selective sweeps and/or background selection) can generate similar patterns of divergence (Cruickshank & Hahn, 2014). These confounding factors limit the potential of simple genome scan approaches, requiring careful interpretation of the main causes of excess *F*_ST_ (e.g. Cruickshank & Hahn, 2014, Tavares et al., 2018). Although advances have been made in *F*_ST_ outlier detection, patterns of differentiation *alone* have limited power to inform us about the current interplay between selection and gene flow along the genome. Recent genomic studies emphasize the role of both linked selection and barriers to gene flow in shaping these patterns. For example, in sea bass (Duranton et al., 2018), regions of low recombination experience faster divergence in allopatry due to selective sweeps, and they also better resist introgression after secondary contact. Moreover, using the geographic context of populations provides a powerful way to distinguish genomic regions harbouring barrier loci. This allows us to overlay patterns of divergence across genomes with the location of loci resisting introgression, such as those displaying steep geographic clines in hybrid zones (Tavares et al., 2018).

In this context, the study of Rifkin et al. (2019) makes an important contribution to our understanding of the mechanisms that generate divergence during the early phase of speciation. Crucially, their work is based on a clever spatial sampling scheme, which includes both allopatric and sympatric populations of two closely related annual weeds (figure 1 in Rifkin et al., 2019): *Ipomoea cordatotriloba* (mixed‐mater) and *Ipomoea lacunosa* (selfer) from the south‐eastern United States. All their analyses are subsequently built on the comparison between allopatric and sympatric populations to control for other factors that may influence interspecific divergence regardless of whether there has been gene flow (Noor & Bennett, 2009). The rationale behind this design is that interspecific barriers should exhibit strong divergence in allopatry and resistance to introgression in sympatry (see Figure 1).

**Figure 1:**
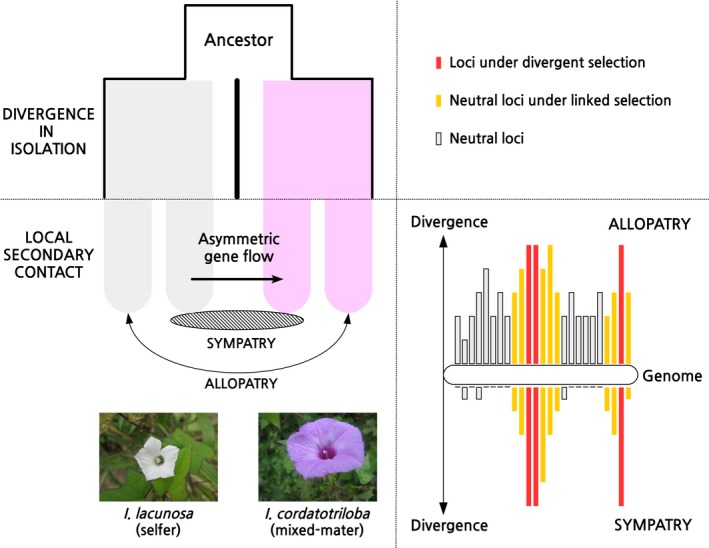
Hypothetical divergence history between the two morning glory species, and resistance to introgression in sympatry. Rifkin et al. (2019) make clever use of the geographic context together with an integrated population genomic toolbox to provide independent lines of evidence that the genomic regions resistant to introgression are also under divergent selection. Photographs courtesy of Irene Liao

Rifkin *et al *.produced transcriptome‐wide SNPs genotyped in 30 *I. cordatotriloba* and 31 *I. lacunosa* accessions. Admixture analyses of 10,873 LD‐pruned SNPs (figure 2 in Rifkin *et al.*) suggest asymmetric introgression from the selfer towards the mixed‐mater. Differential introgression was identified between sympatric and allopatric populations, with introgression more prevalent in sympatry. The authors found concordant results at the phenotypic level (figure 8 in Rifkin *et al.*) with an asymmetric convergence for floral traits in sympatric *I. cordatotriloba* populations. Consistent with previous work (e.g. Brandvain, Kenney, Flagel, Coop, & Sweigart, 2014), this introgression asymmetry towards the mixed‐mater highlights the role of different mating systems in shaping patterns of gene flow in nature. As such, floral traits can be valuable predictors of the direction of interspecific gene flow in plants.

The authors also made a step forward by developing a maximum‐likelihood method to estimate admixture contributions in the presence of selfing. They used this method to validate the above results and also found a higher selfing rate in *I. cordatotriloba* in sympatry. Interestingly, this was accompanied by a reduction in herkogamy (spatial separation of male and female parts). Together, these findings suggest that gene flow may have reinforced reproductive isolation *via* increased selfing rates in sympatry. Further experimental work would be required to determine whether a reduction in hybrid fitness has mediated reinforcement in these two morning glory species.

Rifkin et al. (2019) further compared the interspecific allele frequency differences in sympatry and allopatry at 66,729 SNPs (figure 5 in Rifkin et al., 2019). They uncovered two categories of SNPs in allopatry (less divergent vs. most divergent), which exhibit contrasting patterns of divergence in sympatry. By applying their maximum‐likelihood method, they consistently found that the more divergent SNPs were less admixed in sympatry than the less divergent SNPs in *I. cordatotriloba *(figure 6 in Rifkin et al., 2019), and thus may be considered as candidate barriers to gene flow.

The authors use simulations to provide a second line of evidence that this pattern cannot be explained by neutral gene flow alone. They modelled a simplified history of allopatric populations that suddenly became sympatric and were then connected by a single pulse of unidirectional admixture. This introduced interspecific genomic blocks in the recipient *I. cordatotriloba* species. By fitting the simulations to the observations, they provide convincing evidence that divergent selection is required to explain the lower effective introgression rate of the most divergent SNPs (figure 7 in Rifkin et al., 2019). A particular challenge for this type of simulation approach is accounting for both the demographic history of divergence and indirect selective effects that independently affected the genomes of the two species. Building a null model that explicitly considers these genomic and demographic details in order to test for divergent selection would require further research.

A common challenge with current genomic studies is to provide independent evidence that highly divergent regions actually correspond to regions experiencing strong divergent selection. Valuable insights may come from molecular evolution approaches that can identify the signature of long‐term positive selection. The authors used an extension of the classic McDonald–Kreitman test and found that a substantial fraction (~55%) of mutations were fixed between the two species due to adaptive divergent selection, with the remaining consistent with genetic drift. However, some caveats have to be considered when applying this type of molecular evolution approach in a population genetic context. First, the estimates of the rate of adaptation are expected to be biased if the species diverged recently (Keightley & Eyre‐Walker, 2012). Second, McDonald–Kreitman related tests are insensitive to genetic architectures that involve many barrier loci of small effects, because they are not expected to fix during divergence. In such cases, alternative methods to detect positive selection are recommended (Booker, Jackson, & Keightley, 2017).

In this study, Rifkin et al. (2019) clearly show the benefits of a wise spatial sampling, and the input of molecular evolution approaches to our understanding of genomic divergence and barriers to gene flow. Future progress on this system could include estimating the fitness landscape, which may help build on our understanding of how alternative phenotypes are maintained in sympatry. As the authors suggest, given that the two species often grow in such close proximity (i.e. intertwined), they may occupy alternative adaptive peaks in phenotypic space. A powerful way to address this could rely on relating fitness to phenotypes in the field and identifying the agents of selection. As a conclusive note, we stress the importance of developing multi‐layered model systems for the study of speciation which can tackle the mechanisms and the genetic basis of reproductive barriers to gene flow.

## CONFLICT OF INTERESTS

D.L.F. is an evolutionary ecologist at the University of Vienna interested in population genetics, speciation and hybrid zones, plant mating systems, plant‐pollinator interactions, polyploidy and conservation biology. C.F. is an evolutionary geneticist at the Institute of Science and Technology Austria interested in population genetics, molecular evolution, speciation and sex chromosomes.
